# Contemporary Outcomes of Robot-Assisted Partial Nephrectomy: Results from Two European Referral Institutions

**DOI:** 10.3390/cancers17132104

**Published:** 2025-06-23

**Authors:** Francesco Barletta, Nicola Frego, Mario de Angelis, Stefano Resca, Marco Ticonosco, Enrico Vecchio, Sara Tamburini, Alessandro Pissavini, Andrea Noya Mourullo, Bin K. Kroon, Geert Smits, Bernke Papenburg, Edward Lambert, Frederick D’Hondt, Ruben De Groote, Peter Schatteman, Alexandre Mottrie, Geert De Naeyer

**Affiliations:** 1 Unit of Urology, Division of Oncology, Gianfranco Soldera Prostate Cancer Lab, IRCCS San Raffaele Scientific Institute, 20132 Milan, Italy; deangelis.mario@hsr.it; 2 Department of Urology, AZORG, 9300 Aalst, Belgium; nicola.frego@gmail.com (N.F.); stefano.resca95@gmail.com (S.R.); marco.ti151992@gmail.com (M.T.); enrico.vecchio.5@gmail.com (E.V.); sara.tamburini3@studio.unibo.it (S.T.); alessandro.pissavini@gmail.com (A.P.); edward.lambert@azorg.be (E.L.); frederiek.dhondt@azorg.be (F.D.); ruben.de.groote@azorg.be (R.D.G.); peter.schatteman@azorg.be (P.S.); alex.mottrie@azorg.be (A.M.); geert.de.naeyer@azorg.be (G.D.N.); 3 ORSI Academy, 9090 Melle, Belgium; 4 Department of Urology, University of Modena and Reggio Emilia, 41121 Modena, Italy; 5 Department of Urology, IRCCS Ospedale Policlinico San Martino, University of Genova, 16132 Genoa, Italy; 6 Division of Urology, IRCCS Azienza Ospedaliero-Universitaria di Bologna, 40138 Bologna, Italy; 7 Department of Urology, Hospitales Universitarios San Roque, 35001 Las Palmas de Gran Canaria, Spain; 8 Department of Urology, Rijnstate Hospital, 6815 AD Arnhem, The Netherlands; bkroon@rijnstate.nl (B.K.K.); gsmits@rijnstate.nl (G.S.); bpapenburg@rijnstate.nl (B.P.)

**Keywords:** kidney cancer, partial nephrectomy, robotic surgery, surgical experience, perioperative outcomes

## Abstract

**Introduction:** Available guidelines recommend performing nephron-sparing surgery in selected renal cell carcinoma (RCC) patients. Many studies provided robot-assisted partial nephrectomy (RAPN) functional and oncological outcomes, with most of these including a wide timespan and a number of surgeons with different experiences, which might lead to the heterogeneity of the results. In this study, we aim to provide a contemporary report of RAPN patient outcomes performed at two referral centers by experienced surgeons. **Materials and Methods:** Overall, 333 RAPN patients treated at two European referral centers between 2019 and 2021 were identified. Continuous and categorical variables were reported using medians and proportions. Multi-variable logistic regression (MLR) models were fitted to test predictors of off-clamp technique use and trifecta achievement. **Results**: The median age was 65 (IQR: 57–73) years. The clinical stage distribution was as follows: 224 (67%) cT1a vs. 89 (26%) cT1b vs. 20 cT2 (7%). The median warm ischemia time was 14 (10–18) minutes, with trifecta being achieved in 74% (n = 240) of patients. In MLR models predicting off-clamp surgery, an increasing R.E.N.A.L. score was independently associated with a lower chance of attempting such a technique (OR: 0.69, *p*-value < 0.001). In models predicting trifecta achievement, both a higher R.E.N.A.L. score (OR: 0.78, *p*-value = 0.007) and the presence of multiple lesions (OR: 0.29, *p*-value = 0.007) were independently associated with lower chances of reaching the outcome. Significant upstaging of chronic kidney disease (CKD) stage was recorded in 9.4% of patients after one year of follow-up. **Conclusions**: We reported the contemporary outcomes of patients treated with RAPN by highly experienced surgeons from two referral centers. This report represents a valid benchmark that could be used for individual patient counseling in the decision-making process.

## Simple Summary

Available guidelines recommend performing nephron-sparing surgery in selected renal cell carcinoma (RCC) patients. In this study, we aim to provide a contemporary report of robot-assisted partial nephrectomy (RAPN) patient outcomes performed at two referral centers by experienced surgeons. A total of 333 RAPN patients treated between 2019 and 2021 were assessed. The median age was 65 (IQR: 57–73) years. Clinical stage distribution was as follows: 224 (67%) cT1a vs. 89 (26%) cT1b vs. 20 cT2 (7%). Trifecta was achieved in 74% (n = 240) of individuals. A total of 24 (7.2%) patients exhibited positive surgical margins. Significant chronic kidney disease (CKD) stage upstaging was recorded in 9.4% of patients after one year of follow-up. The following report represents a valid benchmark that could be used for individual patients counseling in the decision-making process.

## 1. Introduction

Available guidelines recommend performing nephron-sparing surgery in selected renal cell carcinoma (RCC) patients candidates for surgical treatment with the intent to preserve kidney function, compared to a radical approach [1,2,3]. Scosyver et al. [2], analyzing data from the European Organization for Research and Treatment of Cancer (EORTC) randomized trial 30904, reported that among patients receiving a nephron-sparing surgery (n = 268), at a median follow-up of 6.7 years, 64.7% exhibited an estimated glomerular filtration rates (eGFR) < 60, compared to 85.7% among those receiving radical nephrectomy (n = 273), with a difference of 21.0% (95% CI, 13.8–28.3).

Additionally, when feasible, this approach has provided significant benefits in terms of cardiovascular comorbidity and reduced other-cause mortality risk compared to a radical nephrectomy [4,5,6]. For example, Marchioni et al. [5], relying on the Surveillance, Epidemiology and End Results (SEER) database, reported that, among elderly patients (≥75 years) with T1a renal cell carcinoma, after propensity score matching and competing-risks regression, partial nephrectomy (PN) was associated with reduced other-cause mortality risk compared to a radical approach. Notably, no significant differences were recorded in terms of 30-day mortality according to the surgical approach. The beneficial effect of PN over radical nephrectomy in terms of other-cause mortality was further confirmed by Baudo et al. [4], who reported that PN significantly decreased other-cause mortality risk across all ages (≤59 vs. 60 to 69 vs. ≥70 years) among T1a renal cell carcinoma patients. Moreover, a systematic review and meta-analysis [6] including 26 comparative studies of PN and radical nephrectomy, assessing new-onset chronic kidney disease (CKD) or cardiovascular outcomes, reported that PN was associated with the reduction in new-onset CKD compared to radical nephrectomy with a pooled hazard ratio of 0.27. This beneficial effect was still observed among patients with renal masses with a diameter larger than 4 cm and a pooled hazard ratio of 0.35. Additionally, Capitanio et al. [7] reported that, among patients with cT1a–T1b renal mass and normal renal function before surgery (defined as GFR ≥ 60), PN was independently associated with the lower risk of experiencing cardiovascular events defined as coronary artery disease, cardiomyopathy, hypertension, vasculopathy, heart failure, dysrhythmias, or cerebrovascular disease not known before surgery.

To date, no strong recommendation has been provided regarding the PN surgical technique; the choice to perform a laparoscopic, robotic-assisted (RAPN), or open partial nephrectomy is mainly based on surgeon experience. In this context, Bravi et al. [8], using on a large multi-institutional dataset (RECORd 2 Project, a prospective, observational project promoted by the Italian Society of Urology) that encompassed data from 2331 patients treated with partial nephrectomy for cT1 renal masses via open, laparoscopic, or robotic techniques, reported that the robotic approach was associated with higher rates of trifecta achievement in patients exhibiting renal masses with a PADUA score < 10, compared to the laparoscopic and open approaches However, this beneficial effect was limited when assessing the outcomes of patients with more complex renal masses.

Many studies provided functional and oncological outcomes after RAPN [8,9,10]. However, most of these reports included a wide timespan and surgeons with different experiences, which might lead to heterogeneity when reporting the above mentioned outcomes [11,12]. Indeed, it is well known that factors such as the learning curve phenomenon could impact functional and cancer control outcomes after surgery.

To overcome this issue, we aim to provide a contemporary report of post-operative RAPN outcomes performed at two referral centers by experienced surgeons, with the aim of providing an effective benchmark for this specific technique which could help physicians for counseling individual patients.

## 2. Materials and Methods

### 2.1. Study Population and Variable Definition

A prospectively maintained database (ethical committee approval number: 2023/010) of 359 patients treated with RAPN between 2019 and 2021 was assessed [13]. These procedures, performed using both the transperitoneal and the retroperitoneal approaches [14,15] and using the da Vinci X and Xi robotic systems (Intuitive Surgical, Sunnyvale, CA, USA), were carried out by surgeons with extensive experience—each with more than 100 RAPN procedures performed at the beginning of the data collection (A.M., G.D.N., R.D.G., G.S., B.K.) [16]. The surgeries were conducted at two tertiary care European centers, where more than 100 robot-assisted surgeries are performed every year (AZORG, Aalst, Belgium; Rijnstate Hospital, Arnhem, The Netherlands). Patients with missing information regarding cT stage (n = 26) were excluded from the analyses, resulting in a final population of 333 cT1-2 patients treated with RAPN. Data for demographic variables, such as patient age (years), gender (male/female), and Body Mass Index (BMI; kg/m^2^), were extracted for every patient. Clinical variables were also extracted such as lesion side (left vs. right), number of lesions (1 or >1), and clinical T stage (cT1a vs. cT1b vs. cT2). The renal lesions’ anatomical complexity, classified with R.E.N.A.L. nephrometry score [16,17], was assessed. Renal function was assessed preoperatively, after one year of follow-up using CKD stages [18]. Rates of CKD upstaging was defined as follows: from class I–II to III–V, class III to IV–V, or class IV to V. Data for operative factors, such as estimated blood loss (EBL; 0–250 vs. 251–500 vs. >500 mL), total console time (minutes), and warm ischemia time (WIT; minutes) were assessed as well. Complications were reported according to the Clavien–Dindo classification (≤2 vs. ≥3a) [19]. Trifecta was defined as: WIT ≤ 25 min, no positive surgical margin and complications Clavien–Dindo ≤ 2 [19].

All data were collected in strict compliance with the ethical guidelines outlined in the World Medical Association’s Declaration of Helsinki pertaining to human subjects in medical research.

### 2.2. Statistical Analysis

This is a retrospective multi-center cohort study. Continuous and categorical variables were reported using medians with interquartile range and proportions. Multi-variable logistic regression (MLR) models were fitted to test predictors of off-clamp technique, covariates consisted of BMI (continuously coded), R.E.N.A.L. nephrometry score (continuously coded), preoperative CKD stage (1 vs. 2 vs. ≥3), and age at RAPN (continuously coded). The same methodology was repeated to test for predictors of trifecta achievement after surgery. Here, covariates consisted of: BMI (continuously coded), R.E.N.A.L. nephrometry score (continuously coded), off-clamp technique and age at RAPN (continuously coded) [20]. Individual trajectories of CKD stages from preoperative assessment and after one year of follow-up were depicted using an alluvial plot [21].

All statistical tests were two-sided, with the level of significance set at *p* < 0.05. Analyses were performed using the R Software Environment for Statistical Computing and Graphics (R version 4.2.2, R Foundation for Statistical Computing, Vienna, Austria) [22].

## 3. Results

Overall, 333 patients were assessed (Table 1). Median age at the time of surgery was 65 (IQR: 57–73) years. Clinical stage distribution was as follows: 224 (67%) cT1a vs. 89 (26%) cT1b vs. 20 cT2 (7%). A total of 30 patients exhibited multiple kidney lesions. Overall, 27% of patients underwent off-clamp surgery; for the remaining patients, median WIT was 14 (10–18) minutes. Significant blood loss (>500 cc) was reported in 9% (n = 30) of patients, with trifecta being achieved in 74% (n = 240) of individuals. A total of 24 (7.2%) patients exhibited positive surgical margins.

In MLR models predicting off-clamp surgery, increasing R.E.N.A.L. score was independently associated with a lower chance of attempting such a technique (odds ratio [OR]: 0.69, 95%CI 0.59–0.81, *p*-value < 0.001). Conversely, BMI, preoperative CKD stage, and age at surgery did not achieve independent predictor status for performing off-clamp surgery (all *p*-values > 0.05; Table 2).

Subsequentially, MLR models were repeated to test for predictors of trifecta achievement. Here, increasing R.E.N.A.L. score (OR: 0.78, 95%CI 0.06–0.93, *p*-value = 0.007) and the presence of multiple lesions (OR: 0.29, 95%CI 0.12–0.72, *p*-value = 0.007) were independently associated with lower chances of trifecta achievement, while BMI, age at surgery, and use of off-clamp technique were not independently associated with the outcome (all *p*-values > 0.05; Table 3).

**Figure 1:**
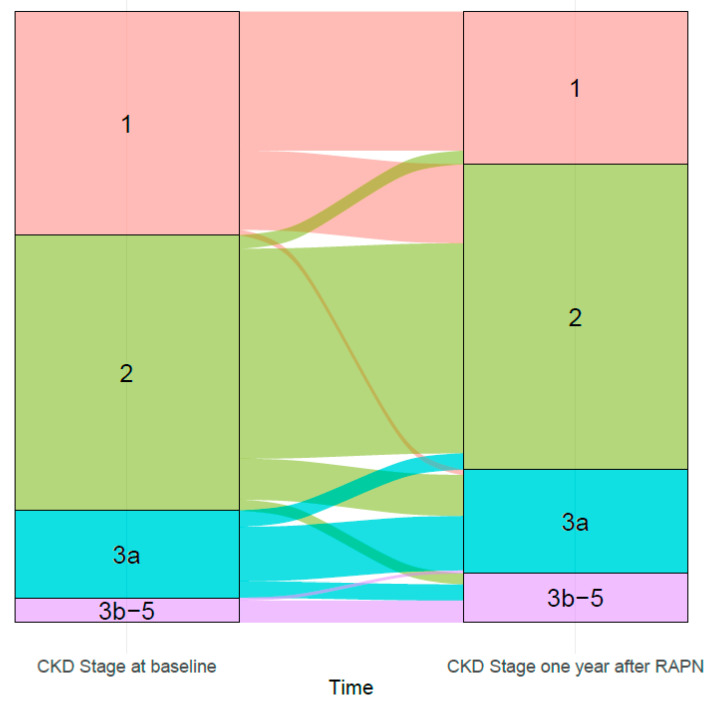
Alluvial plot depicting individual trajectories of CKD stages from preoperative assessment and after one year of follow-up.

At the final pathological evaluation, 84% of patients exhibited malignant lesions. The median hospital stay was 2 (1–3) days. Individual trajectories of CKD stage at diagnosis and at one-year of follow up are depicted in Figure 1, with 83 vs. 13 vs. 4.2% of patients exhibiting CKD 1-2 vs. 3a vs. 3b-5 at diagnosis, and 75 vs. 17 vs. 8.1% of patients exhibiting CKD 1-2 vs. 3a vs. 3b-5 at one year of follow-up after surgery. Significant CKD upstaging was recorded in 9.4% of patients.

## 4. Discussion

In the following report we aimed to assess the outcomes after RAPN in a contemporary cohort of patients treated by experienced surgeons at two European referral centers. Such a report could provide physicians with a valid and reliable benchmark for tailored preoperative counseling of their RAPN patients. Indeed, reports from different data sources might be biased by various confounders (e.g., the inclusion of surgeons with different experiences, a wide time span).

In this context, previous investigators reported that the learning curve significantly impacts on RAPN patients’ outcomes. For example, Larcher et al. [11] reported outcomes of 457 patients with cT1-T2 renal mass and treated with RAPN between 2006 and 2017 at two tertiary care European referral centers. In multi-variable regression analysis, surgical experience was associated with lower WIT, with this relationship being non-linear. Specifically, a steep reduction in WIT was observed from case 1 to case 150, with a significant plateau observed after 150 cases. Additionally, surgical experience was associated with a higher probability of Clavien–Dindo ≥ 2–free postoperative course (OR: 1.03 per 50 cases; CI, 1.01–1.04; *p* = 0.001). This relationship was linear, with no significant plateau reached even after 300 cases.

Similarly, Paulucci et al. [23] aimed at assessing the peri-operative outcomes of 250 consecutive RAPN after the initial learning curve phase, defined as 50 cases. Here, the authors reported that, even after a substantial case load, increasing experience was still associated with better peri-operative outcomes. Specifically, increased experience was associated with the higher likelihood of trifecta achievement (OR = 1.006, *p* < 0.001), with shorter WIT (β = −0.036, *p* < 0.001), less EBL (β = −0.154, *p* = 0.009), less blood transfusions (OR = 0.989, *p* = 0.024), and reduced hospital stay (β = −0.002, *p* = 0.002). It should be noted that increasing surgeon experience was also associated with higher chances of performing RAPN in more challenging cases (e.g., larger tumors) and in patients exhibiting more comorbidity (e.g., hypertension, diabetes and previous abdominal surgery). In consequence, when assessing RAPN outcomes performed by experienced surgeons, a selection bias towards more challenging cases should always be taken into account. This might also explain the positive surgical margin rate of 7.2% reported in the current cohort, which seems slightly higher when compared to other reports. Indeed, Paulucci et al. [23] also reported that increased surgical experience was not associated with the risk of positive surgical margins (β = 0.995, *p* = 0.102).

Our findings are consistent with what was previously reported by other investigators. For example, when comparing our results with those reported by Bravi et al. [8], who analyzed a prospectively maintained database from nine tertiary health care institutions, including patients treated with partial nephrectomy from 2004 to 2018, patients treated with robot-assisted technique (n = 789) exhibited a slightly higher median WIT (15 min) and lower trifecta rate (70%). This difference may be explained by the aforementioned motivations and further validates the need for the current report. These results should also be interpreted considering that Bravi et al. [8] assessed surgical outcomes of only cT1 renal lesions, while in the following report 6% of patients exhibited cT2 disease, reinforcing the concept that reporting outcomes from patients treated by experienced surgeons only is fundamental to provide a valid benchmark for RAPN patients outcomes.

In the current study, we reported a 27% rate of patients treated with the off-clamp technique [24,25]. Notably, in multi-variable analyses, the use of such a technique was not independently associated with an increased risk of not achieving trifecta after surgery. This finding is consistent with what was previously reported by Belmonte et al. [26], which assessed trifecta outcomes in patients treated at one of the centers included in the current study (between 2016 and 2023), according to the use of the off-clamp technique after propensity score adjustment. In consequence, our findings could be interpreted as a validation of the feasibility of performing off-clamp RAPN in selected individuals. Indeed, in multi-variable models, increasing R.E.N.A.L. score was associated with lower chances of performing off-clamp surgery, suggesting that this technique should be attempted in highly selected patients only [27]. It should be noted that in multi-variable analyses, we were unable to adjust to the use of intraoperative ultrasound guidance, which previous investigators found to be associated with improved peri-operative outcomes [28]. These findings are consistent with previous reports, such as Cignoli et al. [29]. Specifically, the authors aimed to assess the association between WIT duration and renal function after nephron-sparing surgery. To do so, the authors relied on a prospective data collection of patients treated with partial nephrectomy at a single high-volume center (n = 1140). Here, the authors reported that patients treated using the off-clamp technique had less complex renal masses, with a significantly lower PADUA score compared to their counterparts treated with on-clamp technique (7 vs. 8, *p*-value = 0.004). Additionally, in multi-variable models predicting functional outcomes, longer WIT was associated with decreased postoperative eGFR, with this relationship not being recorded at 6-month or long-term follow-up. Moreover, off-clamp resection with no ischemia time and partial nephrectomy with short WIT were associated with increased EBL and increased risk of postoperative transfusions. Taken together, these findings confirm that off-clamp surgery should be performed in highly selected patients only, with the benefits of this approach being weighed against the increased risk of intraoperative bleeding.

Additionally, we reported a rate of significant CKD upstaging at one year of follow-up of 9.4% (Table 1). This rate is similar to that reported by Bertolo et al. [9], who assessed RAPN outcomes of a single referral center, with a minimum of 5 years of follow-up after surgery. Notably, the investigators, assessing eGFR continuous values over the study period, confirmed that assessment of kidney function at one year of follow-up resulted as a reliable and strong time point for evaluating the functional outcomes after nephron-sparing surgery, confirming the validity of the endpoint used in the following report [30,31].

Despite the noteworthy results reported, the limitations need to be acknowledged. Our findings must be interpreted within the context of the limitations applicable to observational, retrospective data, in particular when data from multiple surgeons and centers are analyzed.

First, the absence of mid/long-term recurrence data represents the main limitation of this study. However, the aim of the following work was to mainly report RAPN post-operative outcomes, with evaluation of kidney function after one year of follow-up representing a valid endpoint to assess RAPN functional outcomes. Second, continuous values for eGFR were not available; however, categorization according to CKD stages represents a well-established methodology for reporting kidney function after surgery. Third, the same limitation applies to EBL values, which were categorized as 0–250 vs. 251–500 vs. >500 mL. Fourth, surgical morbidity reported according to the Clavien–Dindo classification was only available as grade ≤2 vs. ≥3a.

## 5. Conclusions

In conclusion, we reported contemporary RAPN patients’ outcomes treated by highly experienced surgeons from two referral centers between 2019 and 2021. Limiting the potential bias introduced by the inclusion of surgeons with limited experience and long-time span, the present report represents a valid benchmark that could be used for individual patient counseling in the decision-making process.

## Figures and Tables

**Table 1:** Descriptive characteristics of 333 patients undergoing robot-assisted partial nephrectomy at two European referral centers.

Variables	Overall (n = 333)
**Age at diagnosis (years)**	
Median (IQR)	65 (57–73)
**R.E.N.A.L. score**	
Median (IQR)	7 (5–8)
**BMI**	
Median (IQR)	27 (24–30)
**Sex**	
Male	233 (70)
Female	100 (30)
**ASA score**	
1–2	261 (79)
3–4	72 (21)
**Tumor side**	
Left	158 (47)
Right	175 (53)
**CKD stage at diagnosis**	
1	83 (25)
2	164 (49)
3a	43 (13)
3b-5	43 (13)
**CT stage**	
1a	224 (67)
1b	89 (27)
2	20 (6)
**Console time (min)**	120 (95–150)
Median (IQR)	
Warm Ischemia time (min) *	14 (10–18)
Median (IQR)	
**Off-clamp technique**	90 (27)
**Blood Loss (mL)**	
0–250	219 (66)
251–500	84 (25)
>500	30 (9)
**Clavien–Dindo >= 3a complication after surgery**	11 (3.3)
**Length of stay (days)**	
Median (IQR)	2 (2–3)
**CKD stage one year after surgery**	
1	86 (26)
2	163 (49)
3a	57 (17)
3b-5	27 (8.1)
**CKD upstaging one year after surgery**	31 (9.4)

* Available for 211 patients treated with on-clamp technique.

**Table 2:** Multi-variable logistic-regression models testing predictors of off-clamp technique.

Predictors	OR (95%CI)	*p*-Value
**R.E.N.A.L. Score**	0.69 (0.59–0.81)	<0.001
**Body Mass Index**	1.1 (0.99–1.1)	0.097
**Age at surgery**	1.02 (0.98–1.04)	0.26
**Chronic kidney disease stage**		
1	Ref.	-
2	1.4 (0.71–2.85)	0.3
3–4	1.8 (0.74–4.6)	0.19

OR: odds ratio; CI: confidence interval.

**Table 3:** Multi-variable logistic-regression models testing predictors of trifecta achievement.

Predictors	OR (95%CI)	*p*-Value
**R.E.N.A.L. Score**	0.78 (0.65–0.93)	0.008
**Body Mass Index**	0.97 (0.92–1.04)	0.5
**Age at surgery**	0.99 (0.97–1.02)	0.6
**Number of lesions**		
1	Ref.	-
>1	0.3 (0.12–0.7)	0.07
**Off-clamp surgery**	1.08 (0.49–2.4)	0.9

OR: odds ratio; CI: confidence interval.

## Data Availability

The raw data supporting the conclusions of this article will be made available by the authors on reasonable request.
